# A Pilot Study of Safer Radiation Dosage to the Heart and Its Subregions

**DOI:** 10.3390/medicina57040320

**Published:** 2021-03-31

**Authors:** Rita Steponavičienė, Justinas Jonušas, Romualdas Griškevičius, Jonas Venius, Saulius Cicėnas

**Affiliations:** 1 External Beam Radiotherapy Department, National Cancer Institute, Santariskiu Str. 1, LT-08406 Vilnius, Lithuania; 2 Vilnius University Hospital Santaros Klinikos, Santariskiu Str. 2, LT-08410 Vilnius, Lithuania; jonusas.justinas@gmail.com; 3 Medical Physics Department, National Cancer Institute, Santariskiu Str. 1, LT-08406 Vilnius, Lithuania; romualdas.griskevicius@nvi.lt (R.G.); jonas.venius@nvi.lt (J.V.); 4 Laboratory of Biomedical Physics, National Cancer Institute, Baublio 3b, LT-08406 Vilnius, Lithuania; 5 Department of Thoracic Surgery and Oncology, National Cancer Institute, Santariskiu Str. 1, LT-08406 Vilnius, Lithuania; saulius.cicenas@nvi.lt

**Keywords:** NSCLC, IMRT, heart base, pulmonary artery

## Abstract

*Background and Objectives:* The real impact of ionizing radiation on the heart and poorer overall survival for patients with non small cell lung cancer (NSCLC) remains unclear. This study aims to determine the safe dose constraints to the heart’s subregions that could prevent patients’ early non-cancerous death and improve their quality of life. *Methods and Materials:* A retrospective cohort study was performed containing 51 consecutive patients diagnosed with stage III NSCLC and treated using 3D, Intensity-modulated radiation therapy (IMRT), and Volumetric modulated arc therapy (VMAT) radiotherapy. For a dosimetric analysis, these structures were chosen: heart, heart base (HB), and region of great blood vessels (GBV). Dose–volume histograms (DVH) were recorded for all mentioned structures. Maximum and mean doses to the heart, HB, the muscle mass of the HB, and GBV were obtained. V10–V60 (%) parameters were calculated from the DVH. After performed statistical analysis, logistic regression models were created, and critical doses calculated. *Results:* The critical dose for developing a fatal endpoint for HB was 30.5 Gy, while for GBV, it was 46.3 Gy. Increasing the average dose to the HB or GBV by 1 Gy from the critical dose further increases the possibility of early death by 22.0% and 15.8%, respectively. *Conclusions:* We suggest that the non-canonical sub-regions of the heart (HB and GBV) should be considered during the planning stage. Additional constraints of the heart subregions should be chosen accordingly, and we propose that the mean doses to these regions be 30.5 Gy and 46.3 Gy, respectively, or less. Extrapolated DVH curves for both regions may be used during the planning stage with care.

## 1. Introduction

With an increasing number of cases per year, lung cancer is one of the most frequently diagnosed types of cancers worldwide [1,2]. Radiation therapy (RT) plays a vital role in the treatment of such malignancies. More than 50 percent of patients diagnosed with cancer receive RT as a treatment modality while implementing RT in the treatment plan is recommended in a group of patients diagnosed with stage III unresectable Non-Small Cell Lung Carcinomas (NSCLC) [3]. 

Delivering ionizing radiation (IR) to the chest region while irradiating lung tumors poses a risk of irradiation of other organs such as the cardiovascular system and healthy lung. Historically, the importance of a dose to the heart has been neglected because of low survivability from lung cancer and presumptions arising from breast cancer patients that cardiotoxicity manifests decades after exposure to IR [4,5]. 

Nonetheless, the recent multicenter analysis reported an increased survival time of patients diagnosed with non-operable stage III NSCLC with a median overall survival time of 28.7 months [6,7]. Moreover, a recent study by Atkins et al. retrospectively analyzed a large cohort of 748 patients who were diagnosed with locally advanced NSCLC [8]. During the follow-up, 77 patients experienced an adverse cardiac event. They conclude that cardiac exposure to IR is a risk factor for a cardiac event to manifest before cancer-related death. Such notion is encouraged by the study carried out by McWilliam et al., where the authors completed a permutation analysis of chest irradiation looking for a significant dose distribution over a cohort of patients and found out that a highly significant region was located across the base of the heart [9]. Higher dose exposure to this region was linked with worse patient survival. Such finding was confirmed by the investigation conducted by Jang et al. [10]. His group found that increased dose to the left ventricle increases the acute coronary syndrome risk and impacts patients’ survival time diagnosed with NSCLC. Another study by Han et al. argues that the pulmonary artery region plays a vital role in poorer overall survival, especially if V45 of this area is larger than 70% [11]. 

Moreover, the newest literature review on the topic of cardiac toxicity by Banfill et al. reveals a great interest in the subject, but uncertainty remains because of the lack of prospective high-quality studies [12]. 

In the wake of these new insights in the newest literature, we decided to review patients treated in our institution diagnosed with NSCLC. This retrospective study’s main objective was to determine if there is a statistically significant difference between the dose implemented to the heart subregions highlighted in the article mentioned above by Banfill et al. in the group of patients who experienced early fatal endpoint. Few secondary tasks were set if the main one proved to be significant. Firstly, to perform a regression analysis that could statistically significantly describe the critical factors contributing to the increased mortality of treated patients. Secondly, to determine the critical dose to the heart’s chosen subregions, which leads to the higher risk of the fatal endpoint.

## 2. Materials and Methods

A retrospective cohort study was performed containing 51 consecutive patients (42 males, 9 females) diagnosed with stage III NSCLC (IIIA—44 patients, IIIB—7 patients) and treated using 3D (5 patients), IMRT, and VMAT (46 patients) radiotherapy. Patients were admitted between 2018 and 2019 at our institution. All 51 patients were included in retrospective analysis. The median age of patients during the treatment was 67 years. When the data of this investigation was acquired, it was considered to be the latest follow-up point. If patients died before this date, it was considered that he experienced a fatal endpoint for the purposes of following data analysis. 

**Figure 1:**
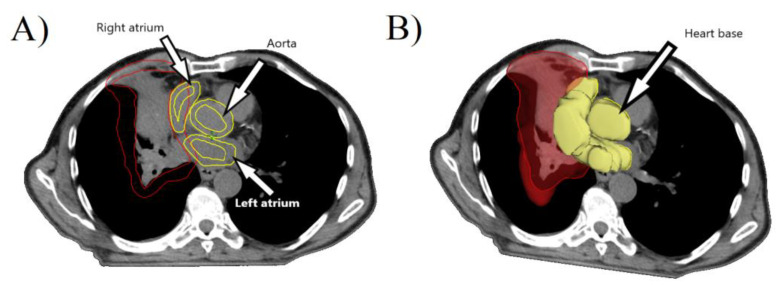
(**A**) Delineation of right atrium (RA), left atrium (LA), and base of the aorta is shown (yellow). Delineated tumor mass is visible (red). (**B**) Formed structure of heart base (HB) is showed (yellow).

42 patients were treated with concurrent or sequential chemotherapy. Standardized chemotherapy schemes were applied using platinum doublet and etoposide. Standard CT and 4D CT scans were obtained before planning. When it was available, PET scans were merged with planning CT scans to delineate tumor mass. Planning CT scan volume extended from the level of the cricoid cartilage to the second lumbar vertebra. Acquired CT scans were with a slice thickness of 2.5 mm. Intravenous (IV) contrast was used for CT scanning, which enables improved delineation of centrally located lung tumours and helps delineate heart structures more precisely. A heart as a critical structure was delineated as recommended by the RTOG contouring atlas. Delineation was done by experienced radiation oncologists and fail-proofed by the independent radiologist. The base of aorta, right atrium (RA), left atrium (LA), and pulmonary artery were delineated as different structures. We have created an inner wall of 4mm separately in RA, LA, which formed a new structure of heart muscle. Finally, Boolean was made of the RA, LA, and the base of the aorta, and new structure of heart base was created (Figure 1A,B). The same was done with the region of great blood vessels (GBV), which consist mainly of the pulmonary artery and base of the aorta.

The median prescribed cumulative dose was 60 Gy (2 Gy/fx), which was delivered to the tumor’s and the metastatic lymph node location while minimizing the dose to the surrounding tissues and organs of interest (spinal cord, lung, esophagus, heart). All patients that were included in the study received and finished the prescribed treatment. 

For a dosimetric analysis, these structures were chosen: heart, the heart base (HB), which consists of the left atrium, right atrium and base of the aorta, and region of great blood vessels (GBV). Dose–volume histograms (DVH) were recorded for all mentioned heart structures. Maximum and mean doses to the heart, HB, the muscle mass of the HB, and GBV were obtained. V10–V60 (%) parameters were calculated from the DVH.

IBM SPSS 26 statistical analysis platform was used to evaluate the obtained data. To find the relation between categorical variables, contingency tables were created, and the chi-square test was used. The normality of continuous variables was checked using a Shapiro–Wilk test. A student *t*-test was used while analyzing data with the normal distribution. The Mann-Whitney U test was performed while analyzing independent data with a non-normal distribution, and the Wilcoxon signed-rank test was used when related samples were compared. Additionally, while analyzing RT relation with fatal endpoint, the Fisher exact test was used. *p*-value was chosen to be 0.05. A logistic regression model was created while analyzing how the previously mentioned dosimetric and demographic parameters (independent variables) influence the increased mortality (dependent variable). Moreover, after receiving statistically significant results, Nagelkerk’s coefficient of determination was calculated, and Hosmer & Lemeshow test was performed, which showed us the regression model’s strength. The increase of probability for the fatal endpoint to occur during follow-up by increasing the mean dose to the HB was obtained from the revised model using Exp (B) parameter, which shows us the odds ratio. The critical dose that will induce the processes leading to the increased risk of death to more than 50 percent of patients after the irradiation of HB and GBV was extracted using a logistic regression graph plot. It is known that in a perfect logistic regression model graph, the curve changes dramatically (the curve is almost perpendicular to the abscises axis) when the probability of the event is equal to 50 percent [13]. The above same knowledge was used in our work. Finally, critical DVH curves with their significant intervals for both regions (HB and GBV) were created using logistic regression models for every dose point from 1 to 70 Gy. For this task, a logistic regression plot was obtained for every DVH dose point. After that, the critical dose was extracted using the model graph as explained before.

## 3. Results

It was found that the entire data except for age and the time after the last RT dose is skewed and kurtotic during the normality check. The Shapiro–Wilk test showed that it is distributed not-normally.

The median age of patients included in this investigation was 67.4 years, ranging from 52 to 83 years. 82.4% of patients were males, and 17.6% were females. 9 patients died during the follow-up time. There was no statistically significant difference between the age of patients who died during the follow-up and those who did not. The mean time after the last RT dose to the follow-up time was 574 days. This cohort was divided into two groups: those who experienced fatal endpoint and those still alive during follow-up. Time after the last RT dose differed statistically significantly (*p* < 0.05). The mean time till endpoint was 320 days for those who died during the follow-up and 629 days for those still alive. Average doses to the heart and HB together with other dosimetric parameters of the heart, HB, and GVB are shown in Table 1. It is worth mentioning that some patients experienced a drop in their arterial blood pressure during the treatment. No need for continuation of antihypertension treatment was needed in this part of the cohort.

After performing the Chi-square test while comparing genders, it was found that there was a statistically significant difference between male and female cohorts in the frequency of fatal endpoint after RT (*p* = 0.028). Male patients experienced fatal events more often in comparison to female patients (9 vs. 0). 

Comparison between patients who suffered fatal endpoints vs. those who survived during follow-up followed (Table 2). There was a statistically significant difference (*p* < 0.05) found when the average doses of the chosen sub-regions of heart and their DVH parameters (V10–V60) were compared between these groups. No other parameter differed significantly among these groups. A clear tendency has been observed—that the HB and the muscle mass of it and GBV of the fatal endpoint group receive a noticeably higher amount of radiation. There was no significant correlation between the chemotherapy usage and the fatal endpoint during the follow-up.

**Figure 2:**
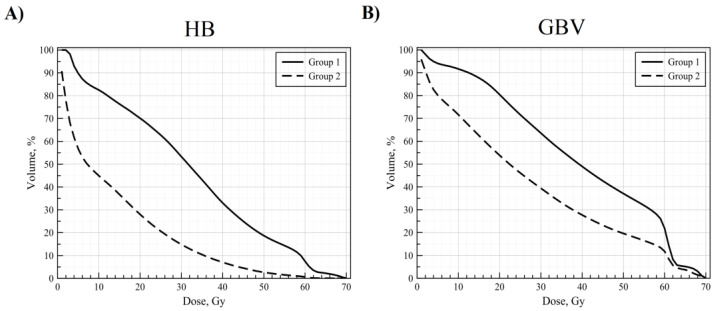
Mean dose–volume histograms (DVH) curves of patients included in the investigation for HB (**A**) and GBV (**B**). Group 1 represents patients who died during the follow-up, while group 2 represents patients who survived during this time.

DVH curves of patients included in the investigation were also compared during the dosimetric analysis (Figure 2A,B). It is visible from the graph that patients who experienced fatal endpoint during follow-up received larger doses to the larger area of the HB and GBV during the treatment. 

After the comparison of the means, two logistic regression models were created for HB and GBV using the following parameters: a dependent parameter was fatal endpoint after the treatment; and the independent parameters were age, gender, maximum and average doses to the heart and HB (or GVB) (Table 3A).

**Figure 3:**
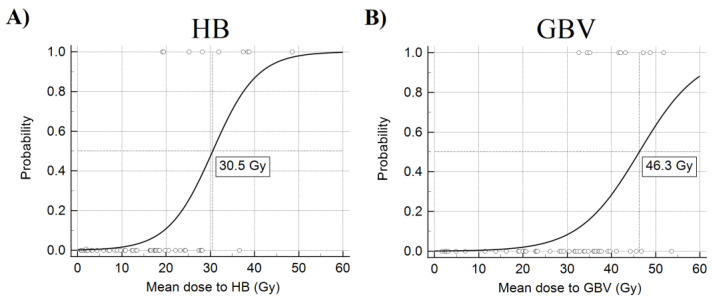
Logistic regression graph plots for HB (**A**) and GBV (**B**). The critical average doses to the heart base and great blood vessels were extracted from these graphs using the above-mentioned analysis methods. The critical dose for HB was 30.5 Gy, while for GBV, it was 46.3 Gy.

Logistic regression analysis showed that only the mean doses to the HB and GBV have a statistically significant impact on the development of a fatal endpoint. Later on, detailed models of logistic regression analysis were created. Calculated Nagelkerke’s coefficients of determination were 0.674 for HB and 0.638 for GBV, showing moderate to strong accuracy of the model. Hosmer & Lemeshow tests significance was well above 0.05 (*p* = 0.959 for HB and *p* = 0.985 for GBV), which indicates that the model is a good fit for the results. After the performed detailed analysis, the average dose of HB and GBV remained statistically significant (Table 3B). Following that, the critical average doses to the heart base and great blood vessels were extracted from the logistic regression graph plots (Figure 3A,B). The critical dose for HB was 30.5 Gy, while for GBV, it was 46.3 Gy. The probability of developing a fatal endpoint after implementing these average doses to the HB or GBV is more than 50 percent. Moreover, the acquired data suggest that increasing the average dose to the HB or GBV by 1 Gy from the critical dose further increases the possibility of death during the follow-up by 22.0% and 15.8%, respectively.

**Figure 4:**
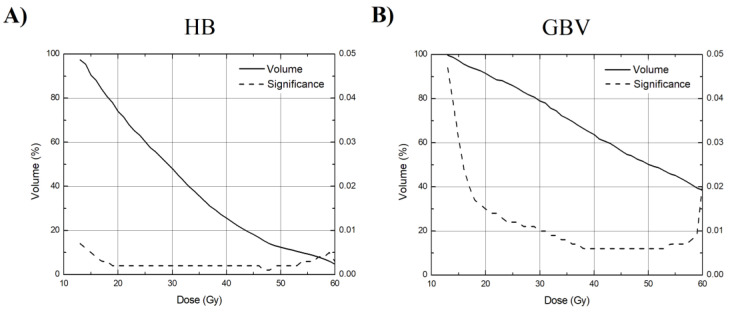
Created critical DVH curves with their significance plotted for HB (**A**) and GBV (**B**). Curves are statistically significant and have a good fit for both subregions in the shown interval from V13 to V60.

Finally, critical DVH curves with their significant intervals for both regions (HB and GBV) were created (Figure 4A,B). Curves are statistically significant and have a good fit for both subregions in the shown interval from V13 to V60. 

## 4. Discussion

Retrospective analysis of patients treated in our institution showed that the heart’s subregions play an essential role in their survival and agrees with the latest literature review in the field [12]. Orientational thresholds of the heart’s subregions that were calculated using the logistic regression analysis for a 50 percent probability of death during the average time of 338 days were 30.5 Gy and 46.3 Gy, respectively. Moreover, increasing the dose to these subregions by 1 Gy increases the risk of the fatal outcome by 22.0 and 15.8 percent, respectively. Furthermore, DVH curves for the heart’s mentioned regions were suggested, which can be used to determine additional constraints for these regions during the planning stage.

There is a prevailing conception among scientists and clinical practitioners that the early death of patients diagnosed with NSCLC is related to radiation-induced adverse cardiac events [14,15]. It was thought that radiation-induced heart disease (RIHD) occurs a decade after RT. This conception is based on studies of breast cancer and Hodgkin lymphoma, where patients are younger, have fewer other morbidities that can affect the overall outcome, and the doses that are being used are much lower [16,17,18]. Meanwhile, patients diagnosed with stage I-III NSCLC receive doses as high as 60–80 Gy, and their survival is expected to be less than 24 months [19,20,21,22]. Moreover, the cardiac dose is not prioritized, and this fact is illustrated by the literature review article by R. Vojtisek, where the author highlights the fact that there are no consistent recommendations regarding dose constraints on the heart and its subregions for the patients diagnosed with the NSCLC [23]. Our results show that concerns related to heart dose constraints should be considered. Patients who died during the follow-up time received statistically significant higher doses to the heart’s substructures containing both atria, base of the aorta, and pulmonary trunk. The same is evident from the inspection of the mean DVH curves (Figure 2)—higher doses were administered to the specific volumes of the HB and GBV in this group. 

Studies report that an increased dose to the heart does increase the possibility for the development of RIHD and reduces overall survival [8,15]. However, as pointed out in a systematic review by Zhang et al., findings reported in the recent scientific literature are inconsistent, and conclusions related to dosimetric parameters cannot be made [24]. Dosimetric parameters used by authors usually are minimum–maximum and mean heart doses and the volume of heart that receives a specific or lesser dose (V30, for example). Unfortunately, there is no regularity concerning which ones are being used. Meanwhile, instead of using the aforementioned classical dosimetric parameters, McWilliam et al. decided to search for a point of dose concentration in the heart, which can be related to RIHD [9]. The dose to the heart base, which exceeds 8.5 Gy, and the maximum dose, which is more than 19.5 Gy, to the region of the right atrium, aortic valve, and right coronary artery was linked with poorer overall survival [12]. Furthermore, when the total dose of more than 63 Gy was applied to the left atrium, a worser OS was observed. OS worsens by 2.2% [8,10,24]. Moreover, if more than 80% of the pulmonary artery’s volume receives a radiation dose of 40 Gy, that lowers the overall survival rate, as well [11]. Mentioned investigations go along with the results published by Hart et al., where the authors show that the peak of the dose shift towards the heart is associated with reduced survival [25]. Our investigation confirms the hypothesis of the articles above and goes along with other articles that are published [10,11,12]. Patients who experienced fatal endpoints during follow-up time received statistically significantly higher doses to the heart’s aforementioned subregions. Moreover, the logistic regression analysis showed that increasing the dose by 1 Gy to the regions of HB and GBV increases the probability of early death by 22.0 and 15.8 percent, with the critical dose being 30.5 Gy and 46.3 Gy, respectively.

Interestingly, the highest separation between the mean DVH curves of the analyzed groups of patients was in the region around this critical dose in the case of HB, indicating that this dose may pose substantial importance to the RIHD and OS (Figure 2A). We suggest that constraints of the heart’s sub-regions should be taken into account during the planning phase more seriously. Our proposed DVH curves may be used for this purpose with great care accordingly (Figure 4). Using these calculations, we assume that increasing the sub-regions volume receiving the dose of ionizing radiation above the proposed threshold (part of the DVH curve where significance is less than 0.05) increases the probability of early death and poorer OS.

The exact mechanism of how ionizing radiation causes the RIHD and affects the overall survival is still not well understood. Considering the whole spectrum of symptoms, it can cause, many factors must contribute to it. As described by Taunk et al., the main endpoint for tissues during the RIHD is microvascular dysfunction and endothelium damage, which leads to fibrosis [26]. Moreover, IR initiates the atherosclerotic mechanisms or enhances those which have already started [27,28]. Finally, a chain of the before-mentioned events leads to the clinical manifestation of coronal artery disease, valvular heart disease, conduction system dysfunction, and other pathologies [29]. Another aspect we must take into account is the structures that are irradiated in the heart. Cardiopulmonary baroreceptors and a sinoatrial node with conduction paths reside in the volume of the heart base. Injury to these structures may lead to pathological changes in ECG and the development of arrhythmias. An investigation by Hotca et al. supports this statement. The authors showed that six months after the dose implementation to the vena cava superior region, which resides in the HB, increases the risk of RIHD (unspecific changes in ECG were observed) [19]. The drop of blood pressure in the group of patients with head and neck malignancies is a well-established fact related to the damage to high-pressure baroreceptors, loss of mass, and dehydration [30]. The aforementioned low-pressure baroreceptors residing in the HB and GBV control the blood volume and, by doing that, control the long-term homeostasis of blood pressure. We stipulate that the drop of blood pressure observed in our patients’ group was caused by the stimulation caused by IR to the low-pressure cardiopulmonary baroreceptors. The acute inflammatory response may cause this stimulation to develop right after the exposure to the IR. This may be explained by the fact that muscle mass, including the endocardium of HB, received statistically significant higher doses in the group of patients with lower OS. The local bioethics committee already warrants future study, where we are planning to investigate the effects of the ionizing radiation to the heart by a differential approach—different structures residing in the region of HB are going to be investigated separately searching for the critical spots there that are responsible for the development of RIHD. 

This study has several limitations. First of all, this is a retrospective study without randomization. Moreover, cardiac events were not documented before and after the treatment. As a result of that, we cannot be sure that the shorter OS of patients who received higher average doses to the HB and GBV are related to adverse cardiac events and RIHD. Second, the patients’ cohort was low in numbers, which limits the statistical methods we can use and the reliability of the ones we used. Also, the increase of immunotherapy usage during the treatment is a rising trend for patients diagnosed with NSCLC. We did not investigate the effects of immunotherapy on the development of RIHD and OS during this investigation. 

## 5. Conclusions

The subregions of the heart play an important role in the overall survival of patients. In agreement with the ongoing trend to reduce the radiation dose that the heart received during RT, this follow-up investigation performed after the publication of the latest literature review by Banfill et al. let us suggest that the constraints of the heart subregions (HB and GBV) should be taken into account and that the mean doses to these regions be 30.5 Gy and 46.3 Gy, respectively, or less. Proposed DVH curves (Figure 4) for both regions may be used during the planning stage with care. 

## Figures and Tables

**Table 1:** Average doses to the heart, HB, and great blood vessels (GBV) together with other dosimetric parameters of the heart, HB, and GVB are shown. Doses received by the muscle mass of HB only are shown in the brackets.

	Min	Max	Median	Range
Mean dose to the HB (Gy)	0.77 (0.80)	48.48 (47.96)	17.26 (17.18)	47.71 (47.17)
Maximum dose to the HB (Gy)	1.79 (1.79)	81.50 (70.54)	60.99 (60.99)	79.71 (68.75)
V10 of the HB (%)	0.00 (0.00)	100.00 (100.00)	56.07 (52.61)	100.00 (100.00)
V20 of the HB (%)	0.00 (0.00)	100.00 (100.00)	33.66 (34.33)	100.00 (100.00)
V30 of the HB (%)	0.00 (0.00)	95.76 (94.03)	15.92 (17.87)	95.76 (94.03)
V40 of the HB (%)	0.00 (0.00)	76.10 (71.48)	6.65 (8.02)	76.10 (71.48)
V50 of the HB (%)	0.00 (0.00)	49.74 (49.14)	1.49 (2.06)	49.74 (49.14)
V60 of the HB (%)	0.00 (0.00)	24.88 (25.02)	0.01 (0.02)	24.88 (25.02)
Mean dose to the heart (Gy)	0.529	62.826	6.53	62.30
Maximum dose to the heart (Gy)	1.442	76.09	62.40	74.65
V10 of the Heart (%)	0.00	88.16	18.93	88.16
V20 of the Heart (%)	0.00	58.49	8.69	58.49
V30 of the Heart (%)	0.00	38.83	3.90	38.83
V40 of the Heart (%)	0.00	23.91	1.91	23.91
V50 of the Heart (%)	0.00	10.36	0.92	10.36
V60 of the Heart (%)	0.00	2.72	0.09	2.72
Mean dose to the GBV (Gy)	1.74	53.60	32.65	51.86
Maximum dose to the GBV (Gy)	12.24	84.08	63.78	71.84
V10 of the GBV (%)	0.41	100.00	92.42	99.59
V20 of the GBV (%)	0.00	95.40	67.35	95.40
V30 of the GBV (%)	0.00	87.76	47.56	87.76
V40 of the GBV (%)	0.00	72.35	32.78	72.35
V50 of the GBV (%)	0.00	61.41	24.20	61.41
V60 of the GBV (%)	0.00	50.38	12.43	50.38

**Table 2:** Comparison of dosimetric parameters between patients who suffered fatal endpoint vs. those who survived during follow-up. Medians of the parameters and their ranges are shown in brackets. Significance and all dosimetric parameters of the muscle mass of HB only are shown in parentheses. Differences that are statistically significant are bold.

	Survived during Follow-Up	Fatal Endpoint	Significance
Mean dose to the HB (Gy)	**13.32 (5.84) (14.30 (35.60))**	**31.93 (29.31) (31.51 (30.18))**	**0.001 (0.001)**
Max. dose to the HB (Gy)	58.95 (79.71) (58.94 (67.62))	63.24 (11.34) (63.24 (11.34))	0.095 (0.070)
V10 of the HB (%)	**43.66 (100.00) (44,12 (100.00))**	**96.26 (63.59) (95.09 (64.72))**	**0.024 (0.021)**
V20 of the HB (%)	**26.44 (89.55) (26.48 (87.58))**	**71.19 (73.13) (71.46 (76.00))**	**0.003 (0.002)**
V30 of the HB (%)	**11.15 (71.80) (11.89 (70.06))**	**51.25 (71.16) (48.98 (72.19))**	**0.000 (0.001)**
V40 of the HB (%)	**3.46 (38.71) (4.14 (36.64))**	**27.92 (66.73) (28.57 (59.90))**	**0.000 (0.000)**
V50 of the HB (%)	**0.75 (14.18) (0.99 (15.37))**	**13.96 (47.67) (15.14 (46.06))**	**0.001 (0.000)**
V60 of the HB (%)	**0.00 (5.85) (0.00 (6.34))**	**4.37 (24.88) (5.71 (25.02))**	**0.002 (0.002)**
Mean dose to the heart (Gy)	5.52 (62.29)	8.04 (21.29)	0.816
Max. dose to the heart (Gy)	62.28 (74.65)	62.59 (41.89)	0.090
V10 of the Heart (%)	14.50 (81.06)	27.02 (83.15)	0.094
V20 of the Heart (%)	7.36 (55.30)	11.65 (58.15)	0.059
V30 of the Heart (%)	3.17 (33.58)	5.86 (38.83)	0.070
V40 of the Heart (%)	1.49 (15.22)	4.02 (23.91)	0.065
V50 of the Heart (%)	0.72 (6.45)	2.04 (10.36)	0.061
V60 of the Heart (%)	0.05 (2.72)	0.16 (2.65)	0.313
Mean dose to the GBV (Gy)	**29.83 (51.86)**	**42.09 (19.11)**	**0.001**
Max. dose to the GBV (Gy)	63.86 (71.84)	63.66 (9.17)	0.913
V10 of the GBV (%)	**83.89 (99.59)**	**100.00 (7.88)**	**0.001**
V20 of the GBV (%)	**62.90 (95.36)**	**90.58 (19.79)**	**0.000**
V30 of the GBV (%)	**41.90 (87.76)**	**71.90 (33.82)**	**0.001**
V40 of the GBV (%)	**28.77 (67.15)**	**51.19 (42.71)**	**0.001**
V50 of the GBV (%)	**21.69 (58.99)**	**38.89 (44.55)**	**0.002**
V60 of the GBV (%)	**12.19 (47.56)**	**23.20 (40.85)**	**0.04**

**Table 3:** Logistic regression models for HB and GBV. Independent variables are underlined. HB and GVB below them refer to the region of interest for which the logistic regression model was created. Age, gender, chemotherapy, average dose to the heart, and maximum dose to the heart were the same during both analyses. In contrast, average dose to the HB and GBV and maximum doses to these regions were unique independent variables and were not included in the analysis of each other (HB was not included in the model of GBV and vice versa). (A) General model for both subregions, showing that only average doses to these sub-regions play an essential role in patients’ survival. (B) Detailed model for both subregions showing that the average dose of HB and GBV remains statistically significant. Parameters that are statistically significant are bold.

**(A) HB: Nagelkerke R Square = 0.674; Hosmer & Lameshow Test: Chi-Square = 2.563; Sig. = 0.959** **GVB: Nagelkerke R Square = 0.638; Hosmer & Lameshow Test: Chi-Square = 1.878; Sig. = 0.985;**				
	**Coefficient**	**Standard Error**	**Sig.**	**Odds Ratio (95% C.I.)**
Age (years):				
HB	−0.036	0.069	0.595	0.964 (0.843–1.103)
GBV	0.063	0.09	0.485	1.065 (0.893–1.270)
Gender:				
HB	−19,474	11,683,949	0.999	0.000 (0.000)
GBV	−23,157	9,436,999	0.998	0.000 (0.000)
Chemotherapy:				
HB	−1.237	1.720	0.472	0.290 (0.010–8.450)
GBV	0.428	1.291	0.740	1.535 (0.122–19.284)
**Average dose to the HB (Gy)**	**0.268**	**0.107**	**0.012**	**1.307 (1.060–1.612)**
**Average dose to the: GBV (Gy)**	**0.352**	**0.170**	**0.038**	**1.422 (1.020–1.982)**
Maximum dose to the HB (Gy)	0.031	0.093	0.735	1.032 (0.860–1.238)
Maximum dose to the GBV (Gy)	−0.024	0.194	0.901	0.976 (0.668–1.428)
Average dose to the heart (Gy):				
HB	−0.124	0.118	0.293	0.884 (0.702–1.113)
GBV	0.007	0.040	0.866	1.007 (0.931–1.089)
Maximum dose to the heart (Gy):				
HB	−0.029	0.041	0.474	0.971 (0.896–1.052)
GBV	0.027	0.052	0.610	1.027 (0.927–1.137)
**(B) HB: Nagelkerke R Square = 0.546; Hosmer & Lameshow Test: Chi-Square = 6.464; Sig. = 0.595** **GVB: Nagelkerke R Square = 0.352; Hosmer & Lameshow Test: Chi-Square = 6.991; Sig. = 0.538**				
**Average dose to the:**				
**HB (Gy)**	**0.199**	**0.064**	**0.002**	**1.220 (1.076–1.385)**
**GVB (Gy)**	**0.147**	**0.056**	**0.008**	**1.158 (1.038–1.292)**

## Data Availability

The data presented in this study are available on request from the corresponding author.
